# Experimental Comparison of Different Carbon Fiber Composites in Reinforcement Layouts for Wooden Beams of Historical Buildings

**DOI:** 10.3390/ma10101113

**Published:** 2017-09-21

**Authors:** Francisco J. Rescalvo, Ignacio Valverde-Palacios, Elisabet Suarez, Antolino Gallego

**Affiliations:** 1 Department of Applied Physics, University of Granada, Campus Fuentenueva s/n, 18071 Granada, Spain; rescalvo@ugr.es (F.J.R.); antolino@ugr.es (A.G.); 2 Department of Building Constructions, University of Granada, Campus Fuentenueva s/n, 18071 Granada, Spain; nachoval@ugr.es

**Keywords:** Timber beams, reinforcement, strengthening, CFRP, rehabilitation, composites

## Abstract

This paper offers a detailed, quantitative and exhaustive experimental comparison in terms of mechanical properties of three different layouts of carbon composite materials (CFRP) used to strengthen existing old timber beams highly affected by diverse natural defects and biological attacks, testing the use of pultruded laminate attached on the tension side of the element (LR), CFRP fabrics totally U-shape wrapping the timber element (UR), and the combined use of both reinforcement solutions (UR-P). Moreover, unidirectional and bidirectional fabrics were considered and compared. Timber elements used for the experimental program were extracted from a recent rehabilitation of the roof of the current Faculty of Law building, University of Granada (Spain), catalogued as a historical edifice. Experimental results from bending tests show that in all cases reinforcement provides a clear improvement in terms of bending capacity and stiffness as compared with the control specimens (without reinforcement). However, improvements in terms of ductility differ considerably depending on the kind of layout.

## 1. Introduction

Reinforcing a structural element basically consists of recovering or increasing its loading capacity. Wood is a natural material commonly used in construction for walls, frames, pillars, beams, trusses, etc. It is generally held to be a very efficient structural material due to its remarkable resistance under tensile and compression loads in relation to its limited density. However, wood has never stood out regarding durability, since it is affected by numerous natural and meteorological factors that can lead to a need for replacement, repair and/or reinforcement with different techniques. This is especially important in historic buildings, where architects usually opt for repair and/or reinforcement rather than total structural replacement.

Traditionally, the reinforcement of wooden structures has involved extra elements, connected with nails, screws and steel, or assembling armor or metal profiles. Metallic reinforcement elements are most often placed on the tension side of the element. Over the years, however, certain limitations of these have become apparent: (1) they increase the weight of the structure; (2) they decrease the height of the place where they will be installed; (3) they have lower durability, mainly due to corrosion processes; (4) they have some geometric limitations, impeding adaptation to non-straight forms of the structure, e.g., arches, vaults or domes, or to the gems of wood elements; and (5) in general, the installation costs are higher. 

Such drawbacks can be alleviated by the use of fiber reinforced plastics (FRP) as reinforcement materials. Their development over the past five decades is remarkable. Reinforcing wood beams using FRP materials is an idea first discussed in the 1960s [1,2,3], where the use of GFRP (fiberglass) was proposed for reinforcement of sawn and glued laminated beams (glulam). In particular, Theakston [1] proposed that if a GFRP fabric is placed wrapped in U-shape to the beam, its ultimate strength and ductility increase considerably. This finding is of enormous importance from the structural safety point of view.

Rowlands et al. [4] put forth the notion of elaborating integrated glulam beams with GFRP embedded in the wood laminate. The authors reported a significant improvement in strength and stiffness provided by the GFRP (respectively, 50% and 20%). This idea is also raised in 1990 by Moulin et al. [5]. 

CFRP (carbon composite) was first applied as reinforcing material in 1992 [6]. In that same year, two important studies [7,8] described how externally glued CFRP pultruded laminates were applied on the tensile side of the element. In the first, the laminate was placed without any prestressing, while, in the second, it was previously prestressed. In the studies of Plevris et al. [7], the results of an analytical model based on the Bazan [9] and Buchanan [10] theory were addressed, through a parametric study of the influence of FRP content and the wood’s mechanical properties, featuring a comparison between CFRP and GFRP. Accordingly, reinforcement produces a change from brittle to ductile failure of the element, and the increase in ductility is acquired with smaller areas of CFRP than of GFRP. The authors indicated that amounts of CFRP material as small as 1% of the cross area manage to increase the strength of the element up to 60%. This value can be further increased when plastification occurs in the compression zone. Similarly, Fiorelli et al. [11] indicated that stiffness increases from 15% to 60%, depending on the amount (from 0.4% to 3%) and type of reinforcing material (GFRP and CFRP) glued in the form of a sheet on the tension side. Shortly thereafter, Valluzzi et al. [12] used CFRP sheets to reinforce wood slabs, obtaining improvements of up to 100% of the ultimate strength under proper humidity conditions (around 12%) yet much lower if installed with a humidity of 22%. Very different results were obtained by Vanerek et al. [13], who used CFRP and GFRP fabrics placed on the tension side, with two thicknesses (0.17 and 1 mm), both in solid and glulam beams; they reported that a CFRP thickness of 0.17 mm was associated with an improvement in ultimate strength somewhere between 2% and 5%, whereas a thickness of 1 mm can attain an improvement between 5% and 40%. They also concluded that when GFRP is used, the improvement achieved is only about of 2–3% and 4–7% for each thickness, respectively. De Jesus et al. [14] compared two pultruded laminated CFRP lengths adhered on the beam’s tension side (350 mm and 600 mm). They concluded that elastic stiffness improved by 23% and 34%, respectively, while the bending load capacity improved by 12% and 28% in each case. In the study by Neubauerová [15], sheets of CFRP were also adhered on the tension side, giving very low improvements in general. In Kim et al. [16], the authors presented experimental results of beams and substructures of spruce wooden beams extracted from a 32-year-old university building reinforced with pultruded CFRP laminate (two sheets) and fabric (six sheets), placed with a progressively decreasing thickness at the ends of the specimens to avoid stress concentrations at these zones, as modeled by Kim et al. [17]. CFRP-strengthening significantly increased the load-carrying capacity of the timber beams from 33% to 184% when compared to the control specimens, with a linear response and no plastification in the strain behavior.

Several works consider the use of reinforcing FRP pultruded rods [18,19,20]. Borri et al. [18] compared the use of externally adhered CFRP pultruded laminates on the tension zone, both on the underside and on the corners of the wooden beams. More precisely, they used one or two rods put into slits in the tension zone. The achieved improvements in ultimate strength were of about 42% and 60% (with two or three sheets, respectively), and 55% with corner sheets, providing in this case a clear improvement in ductility. Improvement in stiffness was between 22% and 30%. At the same time, however, the rods provided a much less ductile behavior than the pultruded laminate and a stiffness increase that did not match the use of laminate (29% with a rod and 52% with two rods). The use of one or two pultruded rods introduced in slots made from the outside, within the tension area, was proposed by Johnsson et al. [19] for glulam beams. The resulting increase in load capacity was between 44% and 63%, and some improvements in ductility were noted, demonstrating the advantages of two rods instead of just one.

Further works have reported results in the case of FRP sheets introduced in slits from the outside [21,22,23,24,25]. In the study of Nowak et al. [23], CFRP sheets embedded in lateral slits in the tensile zone were proposed: three sheets were inserted on each side, and a roughly 21% increase of the flexural capacity was obtained, with a significant increase of the mid-deflection and plastification area of the beam. Nowak et al. [24] extended these results for the case of reinforcement vertically inserted in slits, reporting improvements in the bending load capacity of up to 19%.

Regarding the location of reinforcement, Buell et al. [26] considered four reinforcement layouts, to compare reinforcement only on the tension zone versus sideways wrapping the beam with CFRP fabric, including the compression zone. The improvement varied between 17% and 27% in stiffness, 40% and 53% in flexural strength, and between 36% and 68% in shear strength. The ductility increased substantially thanks to the lateral shear reinforcement. The use of GFRP, CFRP and steel placed on the tensile side, the compression side, or both was compared by Alam et al. [27], results showing that when reinforcement is placed in both sides (tensile and compression), enhanced results can be achieved. They moreover show that CFRP reinforcement is much more effective than that of GFRP. Finally, Moayyed et al. [28] propose reinforcement by means of pre-stressed vertical pultruded laminates embedded in the tensile zone, using only poplar and spruce wooden beams, arriving at a significant improvement in ductility and loading capacity. 

An additional and quite common failure of wooden beams derives from shear stress, in many cases produced by the existence of drying cracks. Several authors have proposed shear reinforcement systems based on sheets arranged on the lateral faces of the element. Triantafillou [29] proposed using lateral CFRP fabric, and demonstrated a high effectiveness of this reinforcement layout. They concluded that improvement is maximized when the fibers are placed in the longitudinal direction and the height of the reinforcing sheets is slightly larger than a particular value, beyond which the failure of the FRP precedes that of the wood. Ajdukiewicz et al. [30] showed an example of historical relevance: the rehabilitation of the largest wooden tower in Poland by means of CFRP sheet patches. Other authors propose the use of pultruded rods for shear reinforcement. For instance, Radford et al. [31] described the effectiveness of rods embedded in epoxy resin within holes made from the bottom to the upper face, demonstrated experimentally. The authors hold that lateral GFRP plates afford a more effective solution, despite the greater visual impact and economic cost. Buell et al. [26] proposed the use of fabric as U-shaped wrapping around the beam, to gain in shear resistance and ductility. Bidirectional CFRP fabric was used and different layouts were compared in their paper, and the lateral reinforcement was found to provide a considerable increase in ductility. When lateral reinforcement is not put in place and only reinforcement in the tension zone was used, horizontal shear failure is a possibility. The above authors also report that it is better to place the wrap in one piece along the length of the beam, than in several pieces with overlaps, perpendicular to the beam. De la Rosa et al. [32] also used U-shaped fabric FRP reinforcement, comparing bidirectional and unidirectional CFRP and basalt, showing improvements when using bidirectional as opposed to the unidirectional option. A good performance of basalt fiber was also reported, as compared with carbon. 

**Figure 1:**
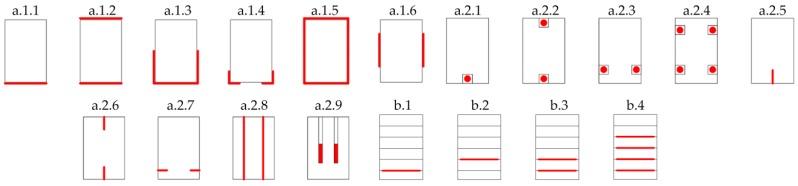
Summary of FRP (fiber reinforced plastics) reinforcement layouts (in red).

Figure 1 offers a general summary of the different layouts of FRP fabrics and pultruded laminates and rods commonly used in previous works. Layouts a.1 correspond to an external positioning of the reinforcement, visible from the outside, while provisions a.2 correspond to slots placed in the element. On the other hand, layouts b correspond to wooden beams reinforced with the element embedded during the laminate process in the case of glulam beams.

However, in most previous research, laboratory specimens were made with freshly harvested wood (new conditions). Few studies have involved wood elements extracted from buildings in service. In the case of historical buildings, due to deterioration over time, the situation can be far different from tests entailing new conditions, as deterioration means a greater number of defects in the wood, hence a greater dispersion in the mechanical properties among specimens extracted from the different structural elements. Although numerous reinforcement layouts have been provided and tested in the past (Figure 1), not many works to date have compared different reinforcement layouts under the same circumstances of the base structural element, as would be the case of old-wood conditions extracted from a single building after many years in service.

In this context, the present contribution offers a comprehensive and experimental comparison of three different layouts of CFRP used for reinforcing timber beams: the use of pultruded laminate strips attached on the tension side of the element (LR), CFRP fabrics totally U-shape wrapping the timber element (UR) and the combined use of both reinforcement solutions (UR-P). Moreover, a comparison between unidirectional and bidirectional fabrics is expounded.

## 2. Material Description

Wood used for these experiments was extracted from 200-year-old beams of the roof of the Law Faculty, University of Granada, during its rehabilitation process. The wood type was *Pinus sylvetris* from southern Spain. Once removed from the roof, the beams were cut and brushed, to obtain a final cross section of (75 ± 1.5) × (145 ± 1) mm^2^ and length 1288 ± 2 mm. The beams were then classified according to the Spanish visual grading standard UNE 56544:2011, which establishes that for beams larger than 70 mm, only two gradings can be assigned: MEG (Madera Estructural de Gran Escuadría) or Rejected. According to standards UNE 338:2016 and UNE 1912:2012, a beam classified as MEG corresponds to the resistant class C18. This paper demonstrates that, although a beam classified as MEG should have better mechanical properties than one classified as Rejected, this is not true in the practice in some cases, mainly due to the presence of extra damage owing to many years in service and the fact that the position of the knots is not taken into account by the standard. Similarly, a beam visually classified as Rejected can, in practice, have higher mechanical properties than one classified as MEG. Out of a total of 51 beams, just 17 were classified as MEG, the most frequent reason for rejection being the presence of slits. Compression and flexural tests were also carried out by using part of these beams for wood characterization, demonstrating very relevant dispersions among beams, not only due to the heterogeneous nature of the old wood but also to the many kinds of defects occurring throughout the service life. A technical data sheet was made for each beam, with the position of all knots, density, dimensions and results from the visual inspection, so as to thoroughly analyze them after the bending tests.

Commercial CFRP reinforcement material supplied by SIKA^®^ and DRIZORO^®^ companies (Madrid, Spain) was used throughout the experimental program. In particular, CarboDur-E 512 and 812 (widths of 50 and 80 mm) pultruded laminate of SIKA^®^ were used, along with COMPOSITE 1405 and 1410 (widths of 50 and 100 mm) supplied by DRIZORO^®^. These widths were cut longitudinally to adapt to the widths of timber beams, and two widths were selected—partial width and full width—to make an experimental evaluation of the influence of reinforcement width so as to save CFRP in practical applications. 

In addition, CFRP fabrics were used in some particular layouts. Their good adaptability and easy application on wood with an irregular shape make them tremendously useful for real situations in which the beam section was not constant along the beam, or was affected by many sorts of defects. In particular, the unidirectional fabric SikaWrap^®^-230 C supplied by SIKA^®^, and DRIZORO^®^ WRAP 200 (unidirectional) and DRIZORO^®^ CARBOMESH 210 (bi-directional) supplied by DRIZORO^®^, were used. From both companies, products with densities as close as possible were selected to ensure a fair comparison between them. Preliminary adherence tests gave evidence that the resins RS-30 of SIKA^®^ and RM-CS of DRIZORO^®^ provided better results. To enhance adherence a primer was also used.

## 3. Specimen Description

**Figure 2:**
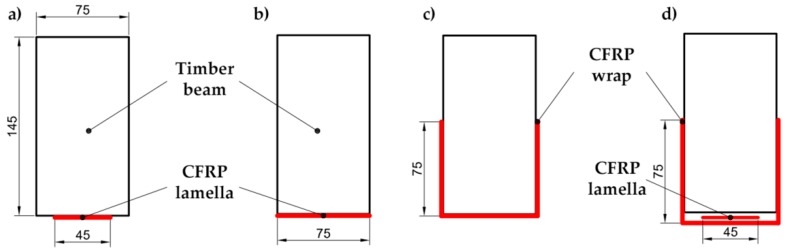
Reinforcement layouts: (**a**) Longitudinal reinforcement, partial width (LR-P); (**b**) Longitudinal reinforcement, full width (LR-F); (**c**) U-shaped reinforcement (UR); and (**d**) Longitudinal and U-shaped reinforcement (UR-P).

Three layouts of reinforcement were considered and compared, as shown in Figure 2. Three specimens were made from each particular layout. LR and UR layouts were selected to compare them with previous works (Cases a.1.1 and a.1.3 of Figure 1, respectively). The UR-P layout, which is a combination of LR and UR ones, is proposed in this paper due to the increase in stiffness provided by the CFRP laminate and, at the same time, the improved shear behavior and greater ductility provided by the CFRP fabric, both increasing the bending capacity of the reinforced element as well. All specimens were reinforced with a single fold for each type of CFRP and layout.

- **Longitudinal reinforcement (LR)**: CFRP pultruded laminate was placed on the bottom side of the wooden beam, considering two different widths: partial, designated LR-P; and full, LR-F.
- **U-shaped reinforcement (UR)**: U-shaped CFRP was emplaced by wrapping the wooden beams. UR nomenclature was used in this case, distinguishing between unidirectional wrap (UR-U) and bidirectional one (UR-B). In both cases, the reinforcement covered the entire length of the beam between the two supports (1000 mm) used in flexural tests.
- **Longitudinal and U-shaped reinforcement (UR-P)**: A combination of two previous layouts was also tested. It basically consisted of a partial-width CFRP pultruded laminate on the tension face covered by a U-shaped fabric reinforcement, and it is referred to as UR-P.

Table 1 summarizes all the specimens made and tested. Non-reinforced beams (NR) were also tested and used as control specimens for comparison reasons. Three beams were tested in each case.

All specimens were conditioned at 20 ± 1 °C and 65 ± 5% relative humidity in a climatic chamber before the tests, to ensure the optimal moisture content. During the reinforcement process, the mean moisture content of the beams was 8.5%. In all cases, application of the reinforcement followed several steps. Firstly, the surface of the beam was prepared before bonding. The beam was sanded and cleaned using a vacuum cleaner. Afterwards, the primer layer was applied, waiting for drying according to the time stipulated on the technical data sheet (curing period of 3–5 days). Once the primer had fully dried, the epoxy resin and the CFRP were applied. Cleaning the CFRP was absolutely necessary in order to guarantee correct adhesion, as indicated by the manufacturers.

## 4. Experiment Description

**Figure 3:**
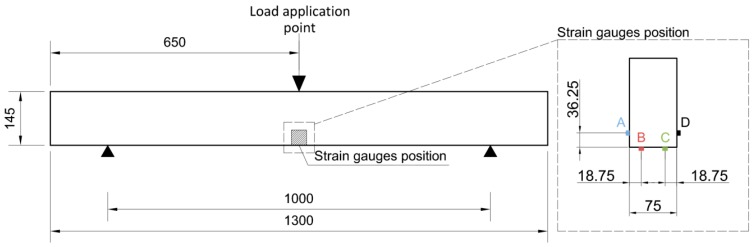
Schematic representation of the 3-point bending test and location of the strain gauges at the beam faces (distances in mm).

The experimental program was carried out through three-point bending tests, as described in Figure 3, applying a continuous load until the final failure of the beam under test. This load was applied with a displacement control of 1.5 mm/min. A CONTROLS S.A. testing machine was used in all cases, model S-110, with an electric actuator having a maximum load of 100 kN. The span between fixed supports was set at 1000 mm. Load and displacement histories were recorded, and compared with the strain measured by four strain gauges attached to the center of the beam, distributed in the zone of maximum tensions: two were placed at the bottom face and two at the lateral faces, as shown in Figure 3.

## 5. Experimental Results

Results are presented for the four groups of beams previously described (NR, LR, UR and UR-P) and divided according to the reinforcement supplier (SIKA^®^ and DRIZORO^®^). Firstly, each section shows the mechanical results obtained for each group of beams, stress against time and stress versus strain results. Subsequently, a comparison is made between the different reinforcement layouts.

As is very well known, wood is a very high heterogeneous material in terms of physical and mechanical properties. Density is one of the physical properties that most affects the ultimate bending capacity (MOR). A high density could be associated with beams having greater amounts of resin or knots in zones of compression, which do not affect bending capacity in the same way. A low density could be associated with beams affected by humidity, large slips or xylophagous insects. For this reason, to make a fair comparison between the different reinforcement layouts, a density-dependent flexural stress correction factor, DC, was considered. It is defined as follows

(1) $$DC=\frac{\rho_{m}}{\rho_{beam}}$$

where $\rho_{m}\;$is the mean density of all specimens subjected to bending tests. Thus, the corrected MOR used for comparison is given by

corrected MOR=DC·MOR,

where MOR is the real one. The DC factor will increase the MOR in a beam with low density, and vice versa. 

**Figure 4:**
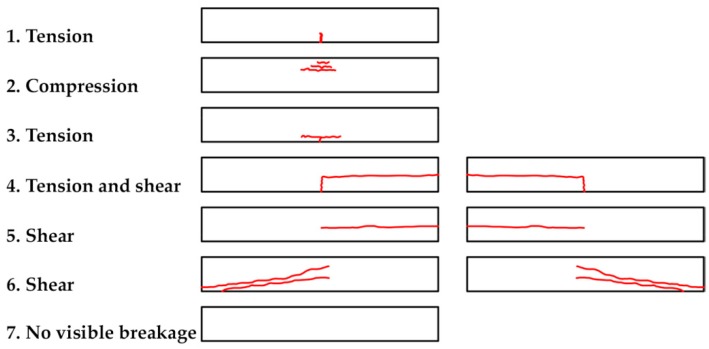
Classification of types of failure.

Fractures in the specimens were classified according to the seven types considered in the studies of De la Rosa et al. [32] and shown in Figure 4.

### 5.1. Non-Reinforced Beams (NR)

**Figure 5:**
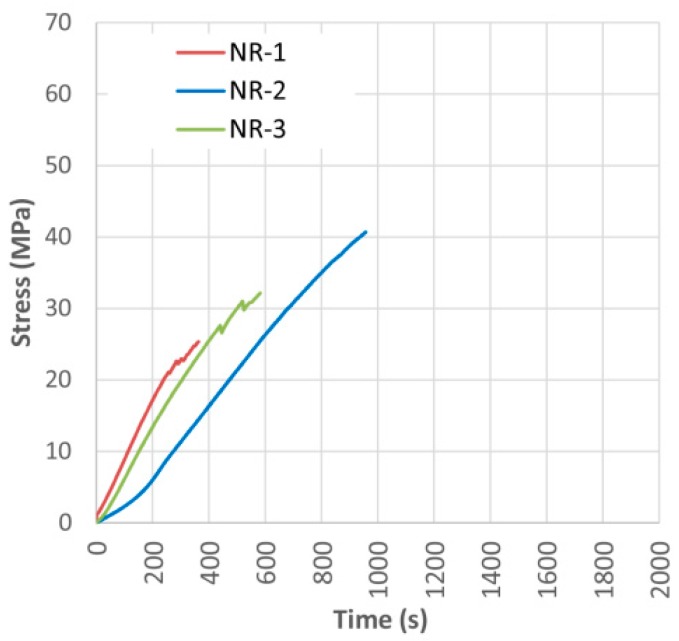
Bending stress over time for non-reinforced beams (NR).

Figure 5 and Table 2 show the bending stress over time, as well as the visual classification, density, maximum bending capacity (MOR), bending elastic modulus (MOE), maximum deflection and type of failure for the case of non-reinforced beams used as control specimens. A clear heterogeneity is observed between the three tested specimens, mainly due to the existence of defects and the different density of the specimens. The observed differences may also derive from the small number of samples used and from the great dispersion of their characteristics. This fact is extensive to all the studied cases, i.e., Section 5.1, Section 5.2, Section 5.3 and Section 5.4, and somehow relativizes the results of this work.

**Figure 6:**
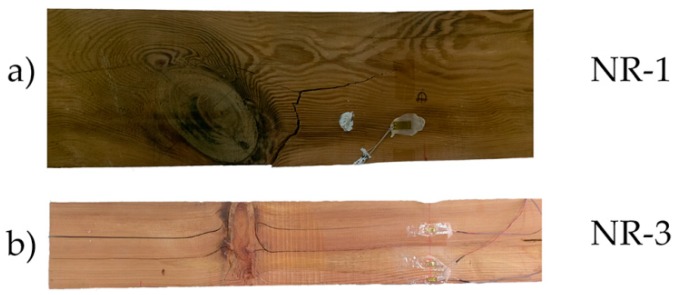
Breakage patterns of the NR beams group: (**a**) NR-1; and (**b**) NR-3.

First, it is seen that the visual classification by itself is not a reliable way to estimate the bending stress capacity of the element. This is basically due to the influence of the position of the knots, an aspect not considered by the visual classification method [33]. For example, one beam classified as rejected, NR-2, had a 29% higher corrected MOR than a beam classified as MEG, beam NR-3. Figure 6 shows that the beams had a brittle breakage, produced by tension or shear stresses. NR-1 beam clearly failed in the tension zone because of the large knot located about 150 mm from the center of the beam. In the case of beam NR-3, the knot was located at the compression zone far from the center, so its effect was not as negative for the bending stress capacity. 

**Figure 7:**
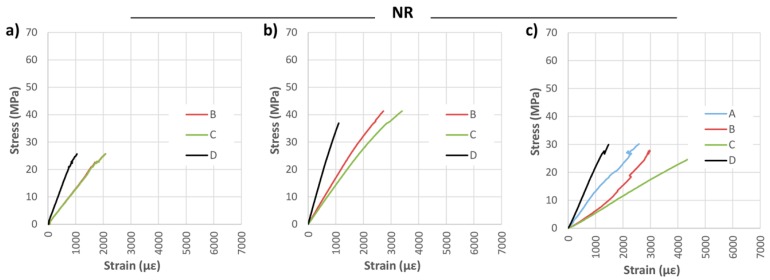
Stress against strain during bending tests: (**a**) NR-1; (**b**) NR-2; and (**c**) NR-3.

Figure 7 shows the bending stress against the strains measured by the strain gauges placed on the specimens, in each case. Clearly seen is a remarkable dispersion in the measured strains, due to defects near the strain gauges and the heterogeneity of the wood. Because of the positions of these defects, the strain gauge—A, for NR-1 and NR-2 beams, could not properly measure the strains. For beam NR-3, despite a low fiber deviation, the measured strains were notably affected by the large knot viewed in Figure 6.

### 5.2. Longitudinal Reinforced Beams (LR)

**Figure 8:**
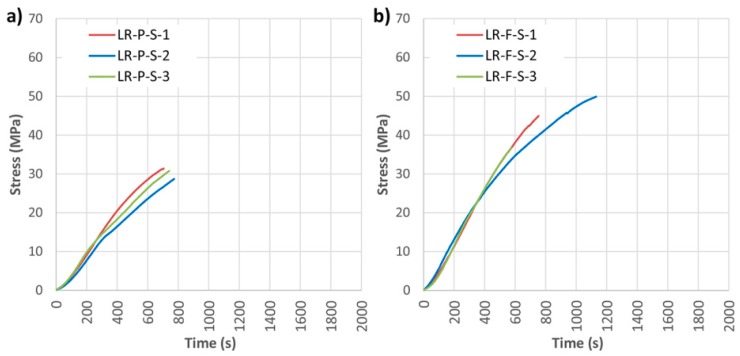
Bending stress against time for longitudinal reinforced beams (LR): (**a**) LR-P-S; and (**b**) LR-F-S. CFRP supplier: SIKA^®^.

Two widths of CFRP, 45 and 75 mm, were considered for the LR layout, named LR-P and LR-F, respectively. As an example, Figure 8 and Table 3 show the mechanical results obtained for the SIKA^®^ products. The table also provides the mean values of each group and the variation in percent with respect to the non-reinforced beams.

It should first be noted that in general the reinforcement in both groups improves mechanical capacities in comparison with non-reinforced beams. For the LR-P-S beams, the effect of the density corrector factor is clear, since the three beams had a density up to 175.76 kg/m^3^ lower than the non-reinforced beams. Thus, the improvement of the corrected MOR with respect to the NR beams was on average 20.35%. Both the MOE and maximum deflection were lower than in the non-reinforced beams, however, mainly due to the presence of woodworm or high defects. 

On the other hand, with LR-F-S an improvement of 36.24% in the corrected MOR is reached (with respect to the beams without reinforcement), as well as improvements of 49.65% and 9.34% for the MOE and maximum deflection, respectively. The LR-F-S-3 underwent a tremendous increase in stiffness, mainly due to its very high density.

**Figure 9:**
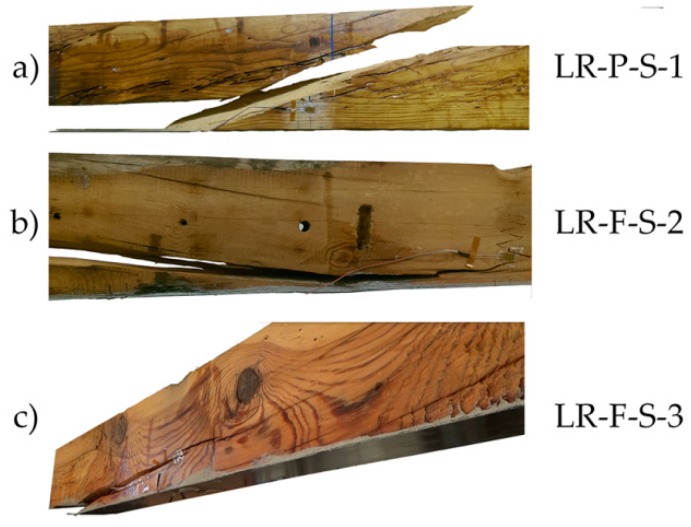
Breakage patterns of the LR beams group: (**a**) LR-P-S-1; (**b**) LR-F-S-2; and (**c**) LR-F-S-3. CFRP supplier: SIKA^®^.

The pattern of failure for LR-P-S and LR-F-S beams was largely due to shear stresses, as observed in Figure 9 for some of the beams of this group. 

**Figure 10:**
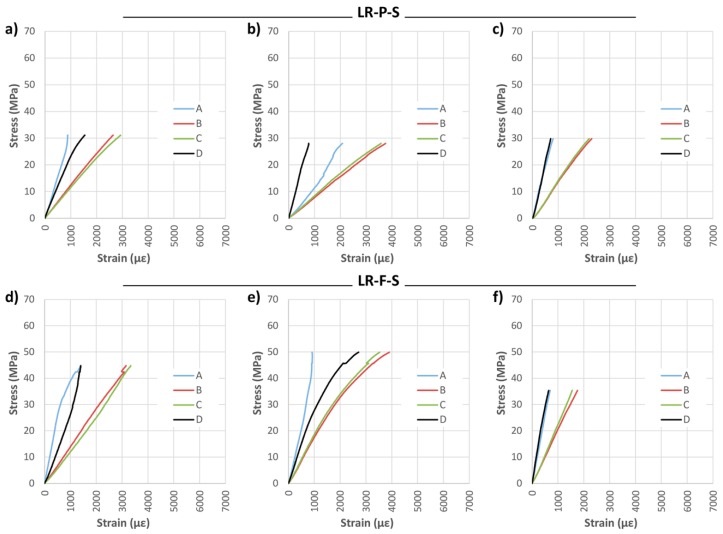
Stress against strain during bending tests: (**a**) LR-P-S-1; (**b**) LR-P-S-2; (**c**) LR-P-S-3; (**d**) LR-F-S-1; (**e**) LR-F-S-2; and (**f**) LR-F-S-3. CFRP supplier: SIKA^®^.

Finally, an analysis of the strain is displayed in Figure 10. A high stabilization of the strain induced by the reinforcement is evident, especially in the lateral strain gauges, placed directly on the wood, despite defects located nearby. The LR-F-S reinforcement provided greater MOR than the LR-P-S reinforcement, at the expense of an increase in stiffness. This means that for the same bending load, the strain difference to be absorbed by the epoxy resin is higher for the LR-F-S layout, weaker against delamination. From the economical point of view, it is important to take into account that the LR-P saves some 40% of reinforcement material when compared with the LR-F layout.

**Figure 11:**
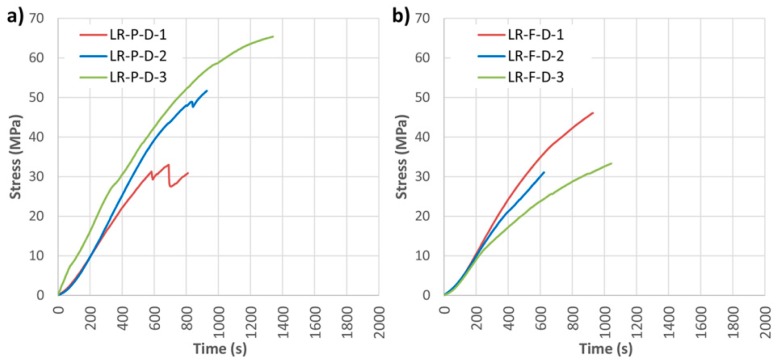
Stress against time for longitudinal reinforced beams (LR): (**a**) LR-P-D; and (**b**) LR-F-D. CFRP supplier: DRIZORO^®^.

Subsequently, the same analysis was carried with DRIZORO^®^ products, named in this case LR-P-D and LR-F-D. Figure 11 shows the results of bending stress versus time for both widths, and Table 4 presents the mechanical results of visual classification, density, maximum bending stress (MOR), elastic bending modulus (MOE), maximum deflection and type of failure.

**Figure 12:**
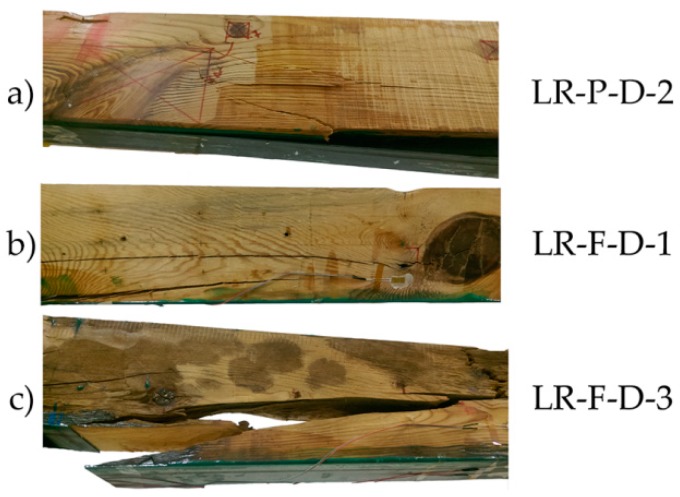
Breakage images of the LR beams group: (**a**) LR-P-D-2; (**b**) LR-F-D-1; and (**c**) LR-F-D-3. CFRP supplier: DRIZORO^®^.

As shown in Figure 11, the bending stress capacity was improved in all cases with respect to the non-reinforced beams. In general, results are similar to the results obtained with SIKA products. The improvement of corrected MOR for groups LR-P-D and LR-F-D with respect to the NR beams was of 42.47% and 33.48%, respectively; MOE improvement was 41.44% and 20.28%, and the maximum deflection improvements amounted to 20.99% and 8.43%, respectively. In the LR-P-S and LR-F-S beams, the type of failure was caused by the shear stress, as shown in Figure 12. Important defects, mainly knots, are observed in all cases. 

**Figure 13:**
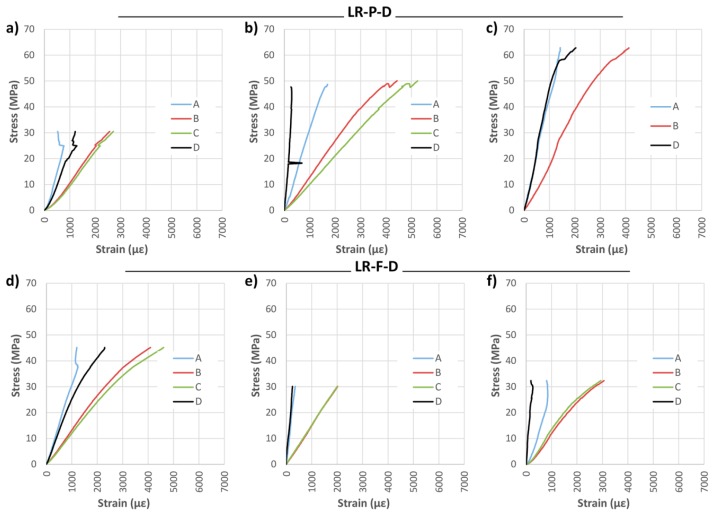
Stress against strain during bending tests: (**a**) LR-P-D-1; (**b**) LR-P-D-2; (**c**) LR-P-D-3; (**d**) LR-F-D-1; (**e**) LR-F-D-2; and (**f**) LR-F-D-3. CFRP supplier: DRIZORO^®^.

Figure 13 shows the stress analysis results. As in the case of LR-P-S and LR-F-S beams, it was observed that the LR-P-D reinforcement (45 mm) provided almost the same maximum bending capacity as for the full-width case (75 mm). In addition, the beams reinforced with the partial width admit higher strains for the same stress, this being a desired behavior against delamination.

### 5.3. U-Shaped Reinforcement (UR)

The use of fabrics to wrap the wood element is of huge practical interest given their easy adaptation to complex shapes, commonly found in the case of old wooden beams. U-shaped reinforcement using CFRP fabric is less often used as reinforcement than LR due to the fact that lateral faces are not always accessible. However, CFRP wrap is used more extensively in the reinforcement of pillars. 

**Figure 14:**
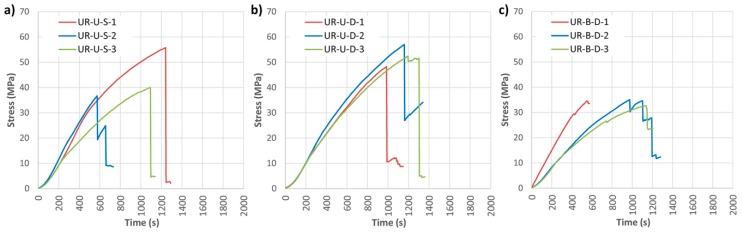
Bending stress against time for U-shaped reinforced beams (UR): (**a**) UR-U-S; (**b**) UR-U-D; and (**c**) UR-B-D. CFRP suppliers: SIKA^®^ and DRIZORO^®^.

Two types of fabrics were considered, unidirectional and bidirectional (UR-U and UR-B respectively). SIKA company did not provide any bidirectional fabric. Figure 14 shows the results of bending stress versus time in both cases, and Table 5 presents the mechanical results indicating the variation with respect to the non-reinforced beams. 

It is clear that, in all cases, the reinforcement improved the bending stress capacity with respect to the NR beams. A ductile behavior is moreover conferred to the wood elements by the CFRP wrap, which translates as a non-fragile failure. This is a major difference with regard to the LR reinforcement layout.

Unidirectional wrap reinforcement (UR-U) improved the mean corrected MOR by 47.16% for SIKA^®^ and 79.15% for DRIZORO^®^ products. The mean MOE did not increase considerably due to the low stiffness contribution of the CFRP fabric to the global stiffness of the reinforced element. In turn, a manifest increase in the maximum deflection was observed (61.72% and 58.54%, respectively, for SIKA^®^ and DRIZORO^®^). In general, the UR-U-S and UR-U-D reinforcements exerted a similar mechanical behavior, with no significant difference found between suppliers of CFRP.

Bidirectional wrap reinforcement did not provide substantial improvement in mechanical properties, giving a corrected MOR increase of 26.97% with respect of the NR beams, and minimal variation in MOE and maximum deflection.

**Figure 15:**
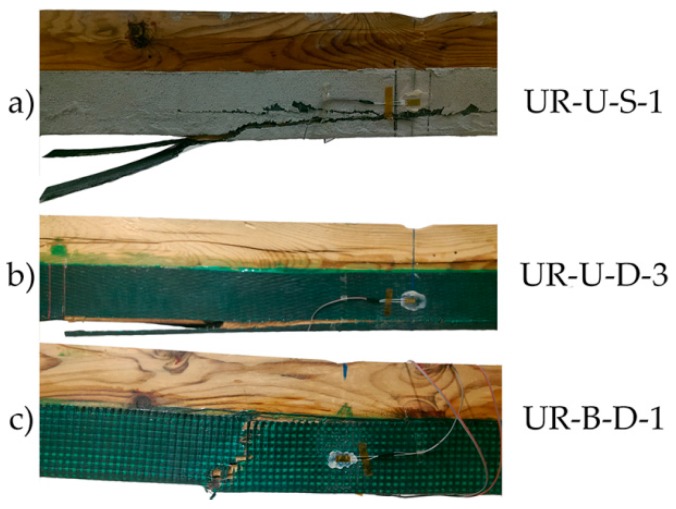
Breakage patterns of the UR beams group: (**a**) UR-U-S-1; (**b**) UR-U-D-3; and (**c**) UR-B-D-1. CFRP suppliers: SIKA^®^ and DRIZORO^®^.

The pattern of failure in all cases was classified as shear one. Some examples are provided in Figure 15.

**Figure 16:**
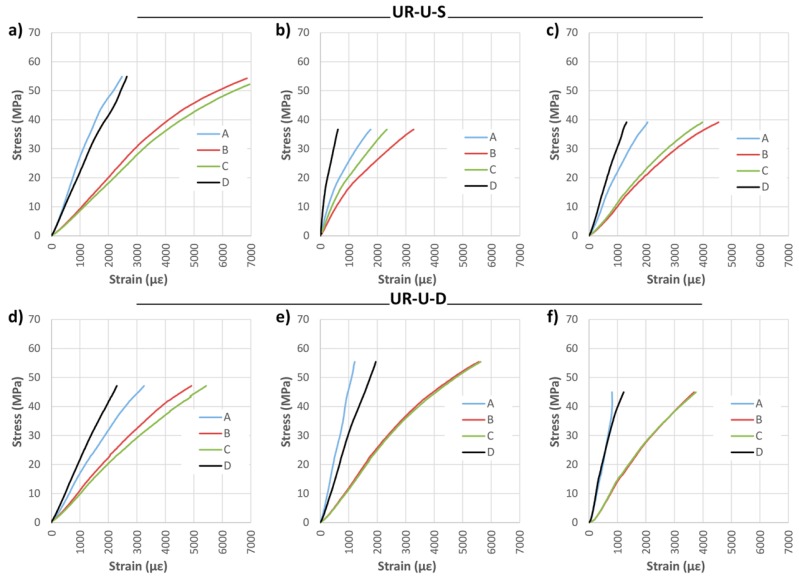
Stress against strain during bending tests: (**a**) UR-U-S-1; (**b**) UR-U-S-2; (**c**) UR-U-S-3; (d) UR-U-D-1; (**e**) UR-U-D-2; and (**f**) UR-U-D-3. CFRP suppliers: SIKA^®^ and DRIZORO^®^.

**Figure 17:**
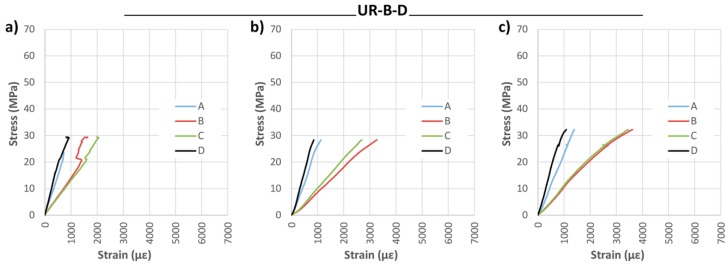
Stress against strain during bending tests: (**a**) UR-B-D-1; (**b**) UR-B-D-2; and (**c**) UR-B-D-3. CFRP supplier: DRIZORO^®^.

Strain analysis is reported in Figure 16 and Figure 17. The great benefit of the CFRP fabric in its adaptation to the strains of the wood is obvious. This behavior holds great interest, because it allows the two materials to work together, meaning the resin does not need to absorb large strain differentials. 

### 5.4. Longitudinal and U-Shaped Reinforcement (UR-P)

**Figure 18:**
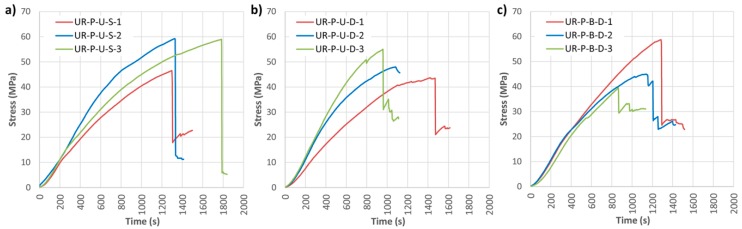
Maximum bending capacity (MOR) against time for U-shaped reinforced beams with CFRP lamella (UR-P): (**a**) UR-P-U-S; (**b**) UR-P-U-D; and (**c**) UR-P-B-D. CFRP suppliers: SIKA^®^ and DRIZORO^®^.

**Figure 19:**
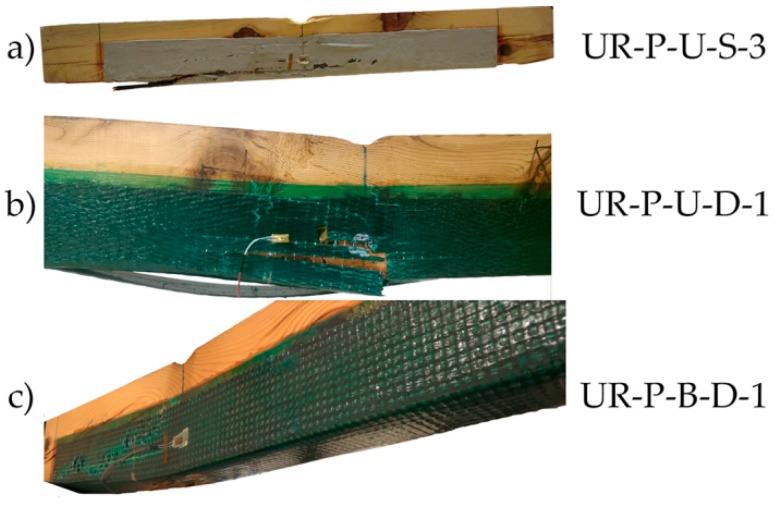
Breakage images of the UR-P beams group: (**a**) UR-P-U-S-3; (**b**) UR-P-U-D-1; and (**c**) UR-P-B-D-1. CFRP supplier: SIKA^®^ and DRIZORO^®^.

Finally, the results of the UR-P reinforcement layout are shown below. This layout consists basically of CFRP partial-width lamella placed at the bottom of the beam covered by a U-shaped fabric. Figure 18 and Figure 19 and Table 6 illustrate the results in this case.

Clear improvements are again observed in terms of the mechanical properties. Furthermore, for this layout, all the beams showed a clearly ductile behavior, confirming the benefits of using CFRP wrap in combination with the CFRP pultruded lamella placed on the tension side of the wood element.

This type of reinforcement also provided a greater stability in the mechanical behavior of the wood elements, with corrected MOR improvements of 58.58%, 47.83% and 77.15% for UR-P-U-S, UR-P-U-D and UR-P-B-D, respectively. The addition of a bidirectional wrap to a wood element with a CFRP pultruded lamella definitively contributes to longitudinal stiffness, allowing a correct absorption of the shear stresses, lending the reinforced element a higher bending stress capacity. The average improvements of MOE were 17.36%, 26.58% and 21.54%, respectively, for the UR-P-U-S, UR-P-U-D and UR-P-B-D beams. Maximum deflection improvements of 74.75%, 54.01% and 33.34%, respectively, were attained. 

Failure mode followed the shear pattern in most cases (Figure 19). Reinforced beams with unidirectional wrap (UR-P-U-S and UR-P-U-D) failed because once the reinforced beam reached a particular stress value, the wrap did not have sufficient capacity to hold the laminate, leading to sudden collapse. Nonetheless, in the case of the reinforced beams with bidirectional wrap (UR-P-B-D), due to the two-dimensional distribution, the fabric not only provided an improvement in shear but also a fastening of the laminate, with a non-clear failure by delamination. 

**Figure 20:**
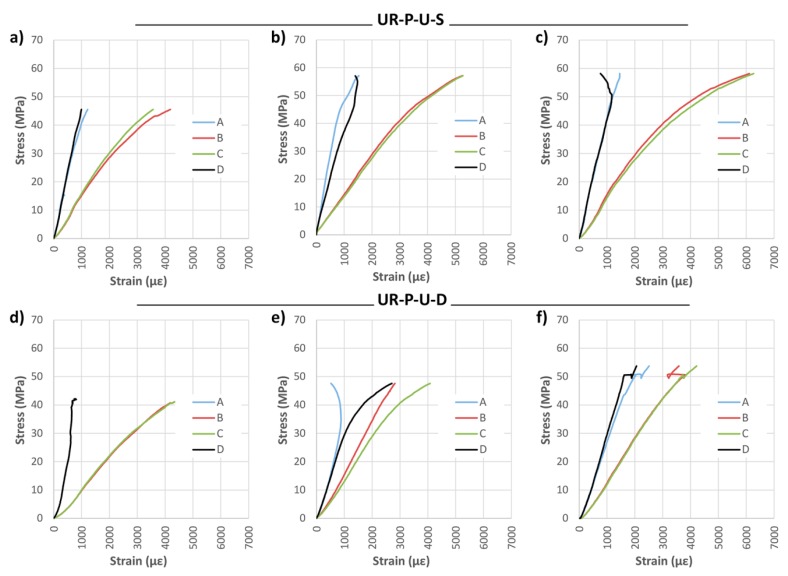
Stress against strain during bending tests: (**a**) UR-P-U-S-1; (**b**) UR-P-U-S-2; (**c**) UR-P-U-S-3; (**d**) UR-P-U-D-1; (**e**) UR-P-U-D-2; and (**f**) UR-P-U-D-3. CFRP supplier: SIKA^®^ and DRIZORO^®^.

**Figure 21:**
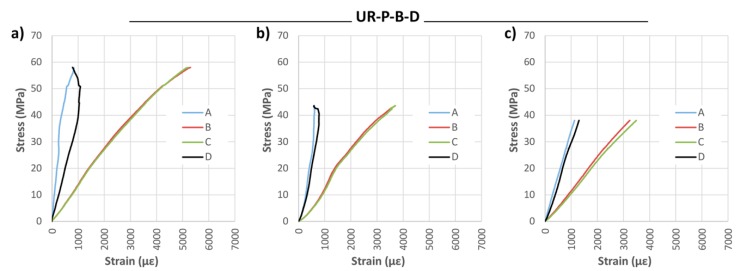
Stress against strain during bending tests: (**a**) UR-P-B-D-1; (**b**) UR-P-B-D-2; and (**c**) UR-P-B-D-3. CFRP supplier: DRIZORO^®^.

Figure 20 and Figure 21 show the results of the strain analysis. A high stiffness and stability is observed in all cases. Moreover, in most of the beams, plastification was found to occur in the mid-span compression zone, which is close to the exhaustion of the maximum compression capacity. This fact has overriding enormous importance in terms of optimizing the use of the wood element. Reinforcement with bidirectional fabric reached strains similar to those of the unidirectional one. 

### 5.5. Comparison between the Different Reinforcement Groups

**Figure 22:**
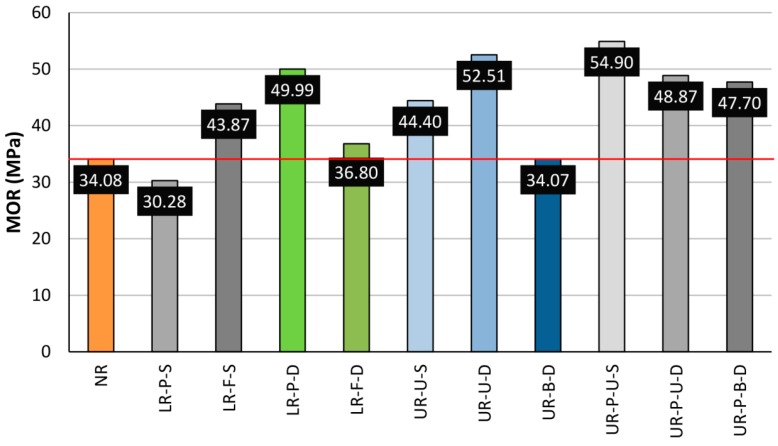
Average MOR for each beam group. Horizontal red line: Average MOR for NR beams.

**Figure 23:**
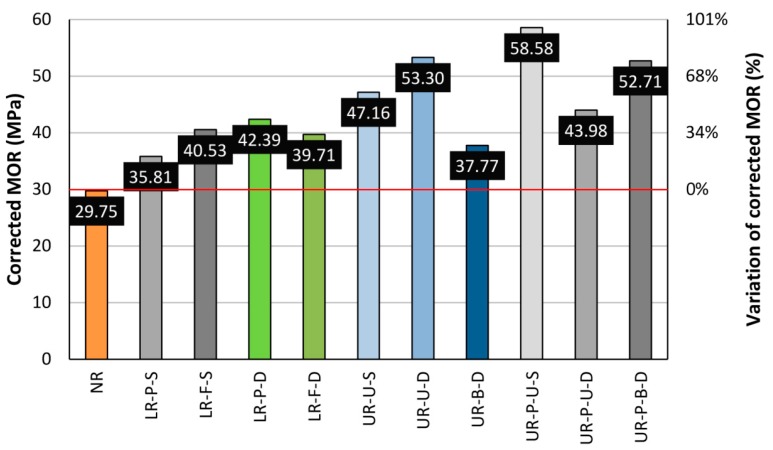
Average corrected MOR for each beam group. Horizontal red line. Average corrected MOR for NR beams.

Once a detailed analysis of the different layouts had been carried out individually, a general comparison was depicted, in Figure 22 and Figure 23, to represent the average MOR and corrected MOR. The horizontal red line indicates the average MOR for the non-reinforced beams.

As deduced from Figure 22, a comparison of different layouts by the mean MOR values, without taking into account any density correction factors, may lead to an incorrect analysis. Figure 23 shows how the introduction of this density correction factor makes the different groups comparable in a more objective and rigorous form. This is due to the major differences in terms of density and defects of each particular beam. In the case of LR beams, a look at Figure 23 shows no great differences between a partial or total width reinforcement. Previous works have stated that the strengthening depends on the area of reinforcing material. However, delamination is the main problem in the case of the LR reinforcement layout. The differences in the strain behavior between wood and CFRP increase the probability of delamination. Resins must absorb these differences when high levels of stresses are reached. As shown in Figure 10 and Figure 13 (stress against strain for LR layouts), the larger reinforcement area for the full-width reinforcement translates as a higher stiffness, and therefore a higher strain difference between the wood and the CFRP lamella. The analysis of this layout showed that increasing the area of reinforcement can produce the effect opposite to the desired one, thus leading to premature failure by delamination, at least in the case of old timber beams. However, for the U-shaped reinforcement, with and without CFRP laminate, the maximum bending capacity would improve with respect to the LR layout, excepting the case of bidimensional fabric (see Figure 23). This type of reinforcement (UR-B-D) provided similar values in conjunction with LR beams. Overall, the UR-P group provides the highest mean MOR values, for both unidirectional and bidirectional fabric.

**Figure 24:**
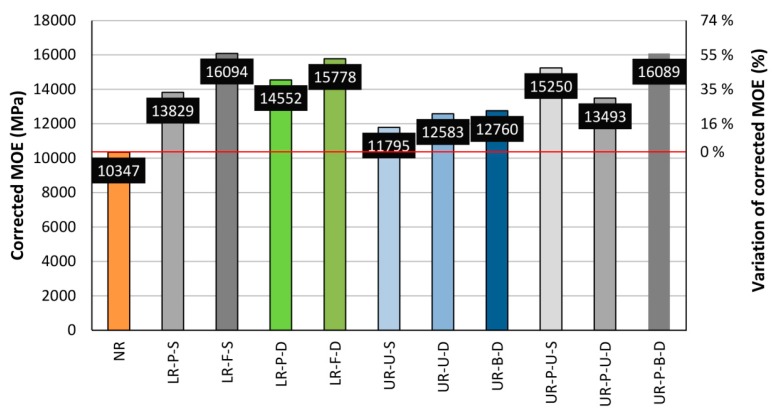
Average corrected MOE for each beam group. Horizontal red line. Average corrected MOE for NR beams.

Figure 24 displays a comparison in terms of the corrected MOE. The corrected MOE was calculated similarly to the corrected MOR, i.e. by means of the DC correction factor. It can be seen that the UR group gave lower values of MOE, but with a relevant 19.64% improvement of the corrected MOE with respect to the NR beams.

## 6. Conclusions

This research provides a quantitative experimental comparison of three different layouts of CFRP used for reinforcing timber beams, which consider the use of pultruded laminate strips attached on the tension side of the element (LR), CFRP fabrics totally U-shape-wrapping the timber element (UR), and the combined use of both these reinforcement solutions (UR-P). Reinforcement improves, in all three cases, the mechanical capacities with respect to a non-reinforced beam (NR). The contribution of the CFRP material is evident, even when the beams showed a high number of defects, knots, woodworm or new and old wood within the same beam section. Visual classification is not a reliable method for determining if a beam classified as MEG will have more bending stress capacity than a rejected beam.

Instead, a new corrective factor is proposed to adjust the ultimate bending stress (MOR) as a function of beam density. 

The average corrected MOR for each beam group shows that both LR-F-S and LR-F-D reinforcement provided greater MOR values than the LR-P-S and LR-P-D reinforcement, but perhaps in exchange for an excessive increase in stiffness. In other words, for the same bending load, the strain difference to be absorbed by the epoxy resin is higher for the LR-F-S layout, having greater weakness against delamination. On the other hand, partial-width longitudinal reinforcement (45 mm) provided nearly the same maximum bending capacity as for the full-width case (75 mm), while continuing to admit higher strains for the same stress, a desired behavior against delamination. 

The use of wrapping fabric to reinforce a wood element is highly appropriate for the complex shapes that an old wooden beam may have. The UR-P reinforcement layout was found to lend the most stiffness and highest stability to wood strains, reaching strain values similar to the UR reinforcement without laminate. Reinforcing beams with CFRP wrap (UR-P) resulted in fabrics with improved shear as well as a fastening of the laminate, and with a non-clear failure by delamination. In addition, most of the beams revealed plastification in the mid-span compression zone, which translates as an almost complete exhaustion of the maximum compression capacity. This finding in itself is enormously important in terms of wood optimization.

Finally, in the case of the UR-P-B-D reinforcement, due to its two-directional distribution, there was a less fragile breaking of the beam. This observation underlines the great potential of such a solution, not only in view of the high contribution of bending stress capacity or the improvements in shear behavior, but also due to the structural security that this type of reinforcement would afford to the base structure. In general, a remarkable heterogeneity in mechanical properties was seen among the three tested samples of each layout. The observed differences may also derive from the small number of samples used and from the great dispersion of their characteristics (due mainly to the existence of defects and the different densities of the specimens). This fact relativizes the results of this work. Regarding approximate costs, UR and UR-P layouts are, respectively, 90% and 230% more expensive than LR. Although this paper focused on old beams, equivalent results could be obtained if non-aged beams were used as the base element, at least for bending capacity improvement. Nevertheless, aged and non-aged beams have a very different behavior in terms of stiffness and ductility, and there would be variability between the samples (less variability in the case of non-aged beams). However, it must be noted that, in real applications, the members in structures are subjected to loads. The structural behavior of the members reinforced by CFRP in existing structures will be quite different than that of the beam tested in this study, because the stress and strain of existing beams are not zero.

## Figures and Tables

**Table 1:** Test matrix for reinforced and non-reinforced timber beams.

Reinforcement Class	CFRP Supplier	CFRP Type	CFRP Lamella Width (mm)	CFRP Thickness (mm)	Fiber Volume Content (%)	Carbon Grammage (g/m^2^)	Nomenclature
Without Reinforcement NR	-	-	-	-	-	-	NR
Longitudinal Reinforcement LR	SIKA^®^	Laminate	45	1.20	70	-	LR-P-S
			75				LR-F-S
	DRIZORO^®^		45	1.40	68	-	LR-P-D
			75				LR-F-D
U-shape Reinforcement UR	SIKA^®^	Unidirectional wrap	-	0.13	-	235	UR-U-S
	DRIZORO^®^	Unidirectional wrap	-	0.11	-	200	UR-U-D
	DRIZORO^®^	Bidirectional wrap	-	0.06	-	210	UR-B-D
U-shape Reinforcement UR-P	SIKA^®^	Laminate + Unidirectional wrap	45	1.20 + 0.13	70	235	UR-P-U-S
	DRIZORO^®^	Laminate + Unidirectional wrap	45	1.40 + 0.11	68	200	UR-P-U-D
	DRIZORO^®^	Laminate + Bidirectional wrap	45	1.40 + 0.06	68	210	UR-P-B-D

**Table 2:** Mechanical results obtained from bending tests of non-reinforced beams (NR). Visual classification, MOR (maximum bending capacity), density, density-corrected MOR, MOE (bending elastic modulus), maximum deflection and type of failure (according to classification of Figure 4).

Name	Visual Classification	MOR (MPa)	Density (kg/m^3^)	Corrected MOR (MPa)	MOE (MPa)	Maximum Deflection (mm)	Type of Failure
NR-1	Rejected	25.74	692.17	21.37	13,410.55	10.39	3
NR-2	Rejected	43.63	653.77	38.35	14,978.33	18.52	4
NR-3	MEG	32.88	639.57	29.54	7508.31	15.21	5
Mean NR value	-	34.08	661.84	29.75	11,965.73	14.71	-

**Table 3:** Mechanical results obtained from bending tests for LR-P-S and LR-F-S beams. Visual classification, MOR, density, density-corrected MOR, MOE, maximum deflection and type of failure. CFRP supplier: SIKA^®^.

Name	Visual Classification	MOR (MPa)	Density (kg/m^3^)	Corrected MOR (MPa)	MOE (MPa)	Maximum Deflection (mm)	Type of Failure
LR-P-S-1	Rejected	31.39	475.80	37.91	11,823.97	13.77	6
LR-P-S-2	Rejected	28.69	485.85	33.93	8417.06	15.71	6
LR-P-S-3	Rejected	30.75	496.58	35.58	14,911.98	10.07	6
Mean LR-P-S value	-	30.28	486.08	35.81	11,717.67	13.18	-
Variation respect NR (%)	-	−11.17	-	20.35	−2.07	−10.36	-
LR-F-S-1	Rejected	44.93	588.21	43.89	14,016.97	14.46	5
LR-F-S-2	MEG	49.96	593.83	48.34	16,890.07	17.97	5
LR-F-S-3	Rejected	36.72	718.25	29.37	22,811.95	15.81	6
Mean LR-F-S value	-	43.87	633.43	40.53	17,906.33	16.08	-
Variation respect NR (%)	-	28.70	-	36.24	49.65	9.34	-

**Table 4:** Mechanical results obtained from bending tests for LR-P-D and LR-F-D beams. Visual classification, MOR, density, density-corrected MOR, MOE, maximum deflection and type of failure. CFRP supplier: DRIZORO^®^.

Name	Visual Classification	MOR (MPa)	Density (kg/m^3^)	Corrected MOR (MPa)	MOE (MPa)	Maximum Deflection (mm)	Type of Failure
LR-P-D-1	MEG	32.94	520.95	36.33	14,095.95	16.39	5
LR-P-D-2	Rejected	51.67	694.63	42.74	12,281.32	20.94	6
LR-P-D-3	MEG	65.36	780.95	48.09	24,397.03	16.05	5
Mean LR-P-D value	-	49.99	665.51	42.39	16,924.77	17.79	-
Variation respect NR (%)	-	46.67	-	42.47	41.44	20.99	-
LR-F-D-1	MEG	46.05	594.35	44.52	13,186.33	15.82	6
LR-F-D-2	MEG	31.06	535.60	33.32	15,616.87	12.99	6
LR-F-D-3	Rejected	33.28	463.09	41.30	14,374.70	19.03	6
Mean LR-F-D value	-	36.80	531.01	39.71	14,392.63	15.95	-
Variation respect NR (%)	-	7.96	-	33.48	20.28	8.43	-

**Table 5:** Mechanical results obtained from bending tests for UR-U-S, UR-U-D and UR-B-D beams. Visual classification, MOR, density, density-corrected MOR, MOE, maximum deflection and type of failure. CFRP suppliers: SIKA^®^ and DRIZORO^®^.

Name	Visual Classification	MOR (MPa)	Density (kg/m^3^)	Corrected MOR (MPa)	MOE (MPa)	Maximum Deflection (mm)	Type of Failure
UR-U-S-1	MEG	55.73	538.45	59.47	10,351.21	25.83	6
UR-U-S-2	Rejected	37.48	546.68	39.39	11,525.73	26.50	6
UR-U-S-3	Rejected	39.99	539.19	42.62	11,474.00	19.02	6
Mean UR-U-S value	-	44.40	541.44	47.16	11,116.98	23.78	-
Variation respect NR (%)	-	30.28	-	58.51	−7.09	61.72	-
UR-U-D-1	MEG	48.26	552.62	50.17	10,373.60	27.78	6
UR-U-D-2	Rejected	56.96	606.37	53.97	13,294.00	21.06	6
UR-U-D-3	Rejected	52.32	539.19	55.75	13,482.09	21.11	6
Mean UR-U-D value	-	52.51	566.06	53.30	12,383.23	23.32	-
Variation respect NR (%)	-	54.06	-	79.15	3.49	58.54	-
UR-B-D-1	MEG	34.53	671.20	29.56	13,502.70	13.14	6
UR-B-D-2	Rejected	35.04	471.19	42.73	10,300.78	14.10	6
UR-B-D-3	Rejected	32.63	456.91	41.03	11,261.17	18.22	4
Mean UR-B-D value	-	34.07	533.10	37.77	11,688.22	15.15	-
Variation respect NR (%)	-	−0.05	-	26.97	−2.32	3.04	-

**Table 6:** Mechanical results obtained from bending tests for UR-P-U-S, UR-P-U-D and UR-P-B-D beams. Visual classification, MOR, density, density-corrected MOR, MOE, maximum deflection and type of failure. CFRP suppliers: SIKA^®^ and DRIZORO^®^.

Name	Visual Classification	MOR (MPa)	Density (kg/m^3^)	Corrected MOR (MPa)	MOE (MPa)	Maximum Deflection (mm)	Type of Failure
UR-P-U-S-1	Rejected	46.52	464.41	57.55	14,867.82	29.95	6
UR-P-U-S-2	Rejected	59.23	529.13	64.32	14,215.19	16.69	6
UR-P-U-S-3	MEG	58.95	628.75	53.87	13,044.72	30.46	6
Mean UR-P-U-S Value	-	54.90	540.76	58.58	14,042.58	25.70	-
Variation Respect NR (%)	-	61.07	-	96.90	17.36	74.75	-
UR-P-U-D-1	Rejected	43.63	542.59	46.21	11,783.48	27.78	4
UR-P-U-D-2	Rejected	47.97	694.86	39.67	17,715.23	22.15	2
UR-P-U-D-3	MEG	55.00	685.90	46.07	15,939.62	18.02	6
Mean UR-P-U-D Value	-	48.87	641.12	43.98	15,146.11	22.65	-
Variation Respect NR (%)	-	43.37	-	47.83	26.58	54.01	-
UR-P-B-D-1	MEG	58.67	521.57	64.64	12,993.80	22.84	6
UR-P-B-D-2	Rejected	44.96	514.03	50.25	17,746.36	20.69	5
UR-P-B-D-3	Rejected	39.47	524.59	43.23	12,888.52	15.30	4
Mean UR-P-B-D Value	-	47.70	520.06	52.71	14,542.89	19.61	-
Variation Respect NR (%)	-	39.94	-	77.15	21.54	33.34	-
